# Perceptions and attitudes about the contribution of the environment to childhood cancer: a pilot study in a medical guild and undergraduate students

**DOI:** 10.1186/s12909-024-05914-0

**Published:** 2024-10-14

**Authors:** Lizet Jarquin-Yañez, Eunice Tello Cruz, Monica Imelda Martinez-Acuña, Jaqueline Calderon-Hernandez

**Affiliations:** 1 Academic Unit of Chemical Sciences, Autonomous University of Zacatecas, Jardín Juárez 147, Centro, Zacatecas, Zac, 98000 Mexico; 2 National Council of Humanities, Sciences and Technologies (CONAHCYT), Insurgentes Sur Avenue # 1582, Credito Constructor, Mexico City, 03940 Mexico; 3 Center for Applied Research in Environment and Health, CIACYT-Faculty of Medicine, Autonomous University of San Luis Potosí, Avenida Sierra Leona No. 550, Lomas 2nd Section, San Luis Potosí, 78210 SLP Mexico; 4 Global Public Health Program, Schiller Institute for Integrated Science and Society, Boston College, 140 Commonwealth Avenue, Chestnut Hill, MA 02467 USA

**Keywords:** Medical perception, Risk factors, Childhood cancer, Environmental medicine

## Abstract

**Background:**

Identifying and recognizing environmental risk factors for childhood cancer is crucial to prevent it. Medical guild are the first contact to monitor children’s health. Therefore, courses about the contribution of chemical toxins in the environment and health outcomes such as cancer should be included in their professional training. This study aimed to evaluate the perceptions and attitudes of a medical guild and undergraduate students in health sciences about the contribution of the environment to childhood cancer.

**Methods:**

A pilot study was conducted, an online survey including thirteen questions was shared among medical guild members and undergraduate students in health sciences. Frequencies, percentages, and chi-square homogeneity tests were calculated to compare groups.

**Results:**

Genetic factors ranked as the first possible cause of childhood cancer (88.2% medical guild and 97.7% undergraduate students). However, 70.6% of medical guild and 64.6% of undergraduate students reported that they have ever suspected that childhood cancer could be related to the environmental conditions in which children live. More than 95% of the participants reported that they would find it useful to have more knowledge about environmental risks and cancer. When data were analyzed by profession (medical guild) and academic year (undergraduate students), no significant differences were observed. Nonetheless, comparisons by academic discipline between undergraduate students, showed that a higher percentage of medicine and environmental sciences and health (over 98%) reported environmental exposure as risk factors associated with childhood cancer compared to 75% from physiotherapy, (*p* = 0.001).

**Conclusions:**

In this study, the environmental contribution to childhood cancer is not clear among the medical guild and undergraduate students. They should be trained on the topic of cancer and the environment.

## Background

Research on childhood cancer has shown that its environmental burden is important, since its etiology includes ionizing radiation and only between 5% and 10% of identified cases are associated with hereditary genetic alterations [1, 2]. This suggests that the rest of the cases could be due to the accumulated and added load of environmental carcinogens present in the places where children develop, even from the prenatal stage [1, 3]. Because children are especially vulnerable to toxic substances present in their daily activities due to their behavioral aspects (playing low to the ground, hand-mouth habits) and their biology (high metabolic rates, developing organs and systems) [4]. Nowadays, children experience widespread and varied exposures to carcinogens from diverse sources and human activities throughout their lives [5–7]. This information is important since these carcinogenic substances are released into the environment where children live, play and go to school through various industrial and anthropogenic activities (smelters, steel mills, petrochemical industry, emissions from motor vehicles, incinerators and the use of biomass to prepare food, among others) [8, 9]. Increased risk of developing some types of cancer, such as acute lymphoblastic leukemia (ALL), acute myeloid leukemia (AML), and Hodgkin’s lymphoma, have been reported in children living close to these emission sources [10–12].

Considering that each geographic area where populations develop (health, industrial, economic, social, etc.) has its own risk scenario, it is necessary to know the approach and resolution that medical guild give to the environmental conditions, since the environment determines the health status of populations. For example, for ALL, the most prevalent type of cancer in children; there are no screening tests or surveillance campaigns for children living in neighborhoods with high burden of carcinogens. This is due to lack of attention to environmental risks, poor understanding and lack of awareness of exposure to toxic substances. So primary health care providers’ perceptions and attitudes about exposure to environmental chemicals and childhood cancer in addition to traditional signs or symptoms, are the key to translating evidence from environmental epidemiological studies into actions to monitor cancer outcomes in children at the primary care level.

The correct identification of risk factors for the contribution of cancer must be based on solid training of medical guild. Since 1995, the National Academy of Medicine of the United States has recommended competencies on environmental health for medical professionals such as: (1) Recognize the signs, symptoms, diseases, and sources of exposure related to environmental agents; (2) Understand the student’s influence of environmental agents on human health; (3) Discuss environmental risks with their patients and provide understandable information on risk reduction [13]. Thus, we designed this research to evaluate the perceptions and attitudes of a medical guild and undergraduate students in health sciences about the contribution of the environment to childhood cancer.

## Methods

### Study design and population

A pilot survey study was designed with two groups of participants: a medical guild from the pediatricians’ college and a group of undergraduate students in medicine, environmental sciences and health, and physiotherapy from San Luis Potosi, located in the north-central region of Mexico.

The surveys were distributed from March to November 2022. The inclusion criteria were being enrolled in university courses in the case of undergraduate students and in the pediatricians’ college in the case of the medical guild and having access to some electronic equipment through which to receive, answer and send the survey.

The design of this study was approved by the Research Committee of the Secretary of Health of the State of Zacatecas (R-17-15 CI 32 056 051-2020/11), and all participants’ free and informed consent was obtained online. In the first section of the survey, the purpose of the study was briefly explained, and the respondents’ consent to participate was obtained.

### Sample size

The sample size was based on the literature referring to other pilot studies where multiple options are presented, including a validated minimum size for questionnaires of 30 and that the sample of a pilot study could be 10% of the projected sample for the more extensive main study [14, 15]. This study considered a sample size 40 for the medical guild and 135 undergraduate students.

### Data collection and measuring instruments

Two instruments were used: an online surveys for the medical guild titled “Medical Perception on the Contribution of the Environment to Childhood Cancer” and another for the undergraduate students titled “Contribution of the Environment to Childhood Cancer.” Both surveys included 13 questions to know the participants’ opinions about the environment’s contribution to childhood cancer.

The procedure to validate the surveys was done by five experts in environmental sciences and health from the Center for Applied Research in Environment and Health (CIAAS-UASLP) who were not directly involved in the survey development. Validation was related to the connection with the study’s objective and the questions’ structure. It consisted of granting a rating on a scale between 1 and 5 to each question for each of the following criteria: connection with the study’s objective, coherence and clarity, and structure and language. The maximum scores for each question and criterion could obtain were 15 and 65, respectively. The validation results showed a minimum average score of 13.8 and 13.1 in the questions for undergraduate students and the medical guild, respectively. And an average score greater than 91.0 in the criteria of both surveys.

The survey was applied voluntarily and anonymously and was distributed online using the QuestionPro© software. The link to the surveys was distributed among the medical guild and the undergraduate students through the pediatricians’ college and the program coordinators of each major/school.

### Statistical analysis

The descriptive statistical analysis included frequencies and percentages, and chi-square tests of homogeneity were conducted to compare prevalence among groups. All analyzes were performed with R© software.

## Results

A total of 34 participants from the medical guild college and 130 undergraduate students accepted the informed consent and completed the survey. The response rate was 85% and 99%, respectively. And the average time it took them to answer the survey was 198 s.

91.2% of the medical guild who answered the survey were pediatricians, 5.9% were pediatric oncologists, and 2.9% were general practitioners (Table 1). 79.2% of the undergraduate students were enrolled in the major of medicine, 14.6% in environmental sciences and health, and the remaining proportion was in physiotherapy (Table 1). When conducting the survey, 46.9% of the undergraduate students were freshmen and sophomores, and the remaining proportion were junior and senior.

**Table 1:** Distribution of participants by classification as medical guild or undergraduate students (in parentheses the sample size)

Medical guild (*n* = 34)	*n* (%)
Pediatrician	31 (91.2)
Pediatrician-Oncologist	2 (5.9)
General practitioner	1 (2.9)
Undergraduate students (*n* = 130)	
Medicine	103 (79.2)
Environmental sciences and health	19 (14.6)
Physiotherapy	8 (6.2)

**Fig. 1:**
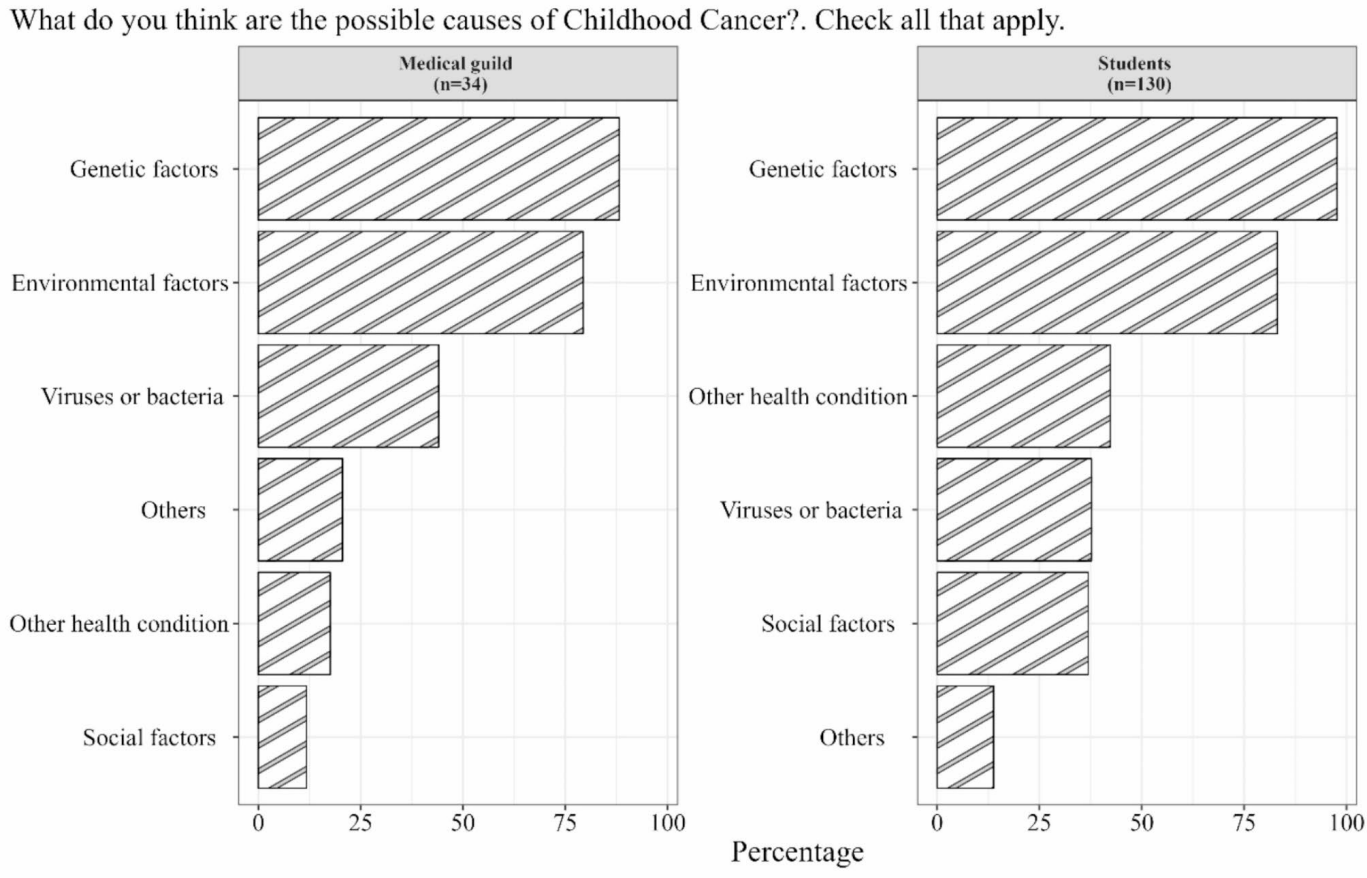
Distribution of responses about the possible causes of childhood cancer (in parentheses the sample size)

Participants were asked about possible causes of childhood cancer. The causes that were most frequently included by the medical guild in descending order were: genetic factors, environmental factors, and viruses or bacteria. And the causes that undergraduate students most frequently included were genetic factors, environmental factors, and other health conditions (Fig. 1).

Analysis of the answers to the questions about whether, in some cases of childhood cancer they have been suspicious of the environment in which the child lives, if they have had or heard doubts about its causes, and if it would be useful for them to have information on the contribution of the environment to cancer children showed the following results: 70.6% of medical guild and 64.6% of undergraduate students have ever suspected that childhood cancer is related to the environment in which the child lives. About 90% of participants in both groups have had or have heard doubts about the causes of childhood cancer. More than 95% of the participants agreed that environmental exposure could be a risk factor for childhood cancer and that it would be helpful for them to have more knowledge about this topic. When data were analyzed by the field of expertise in the medical guild and by years coursed for the undergraduate students, no significant difference was found. However, for undergraduate students grouped by academic discipline, statistically significant differences were found in the question about if environmental exposures may be a risk factor associated with childhood cancer, since a lower percentage of those in physiotherapy (75%) reported that environmental exposure may be a risk factor associated with childhood cancer compared to those in medicine and environmental sciences and health (more than 98%) (Table 2).

**Table 2:** Affirmative responses to selected questions from the survey stratified by academic discipline in the group of undergraduate students (multiple responses)

Question	Medicine	Environmental sciences and health (*n* = 19)	Physiotherapy (*n* = 8)	
	(*n* = 103)			
	n (%)			p-value
1. Have you had or heard doubts about what causes cancer?				
	90 (87.4)	19 (100.0)	7 (87.5)	0.261
2. In any case of childhood cancer have you suspected that it is related to the environment in which the child lives?				
	65 (63.1)	14 (73.7)	5 (62.5)	0.841
3. Could environmental exposures be a risk factor associated with childhood cancer?				
	101 (98.1)	19 (100.0)	6 (75.0)	0.001*
4. Would you find it useful to have more knowledge or information about the contribution of exposure to environmental contaminants and childhood cancer?				
	101 (98.1)	18 (94.8)	8 (100.0)	0.610

*p < 0.05 Chi-square test of homogeneity; n = sample size

**Fig. 2:**
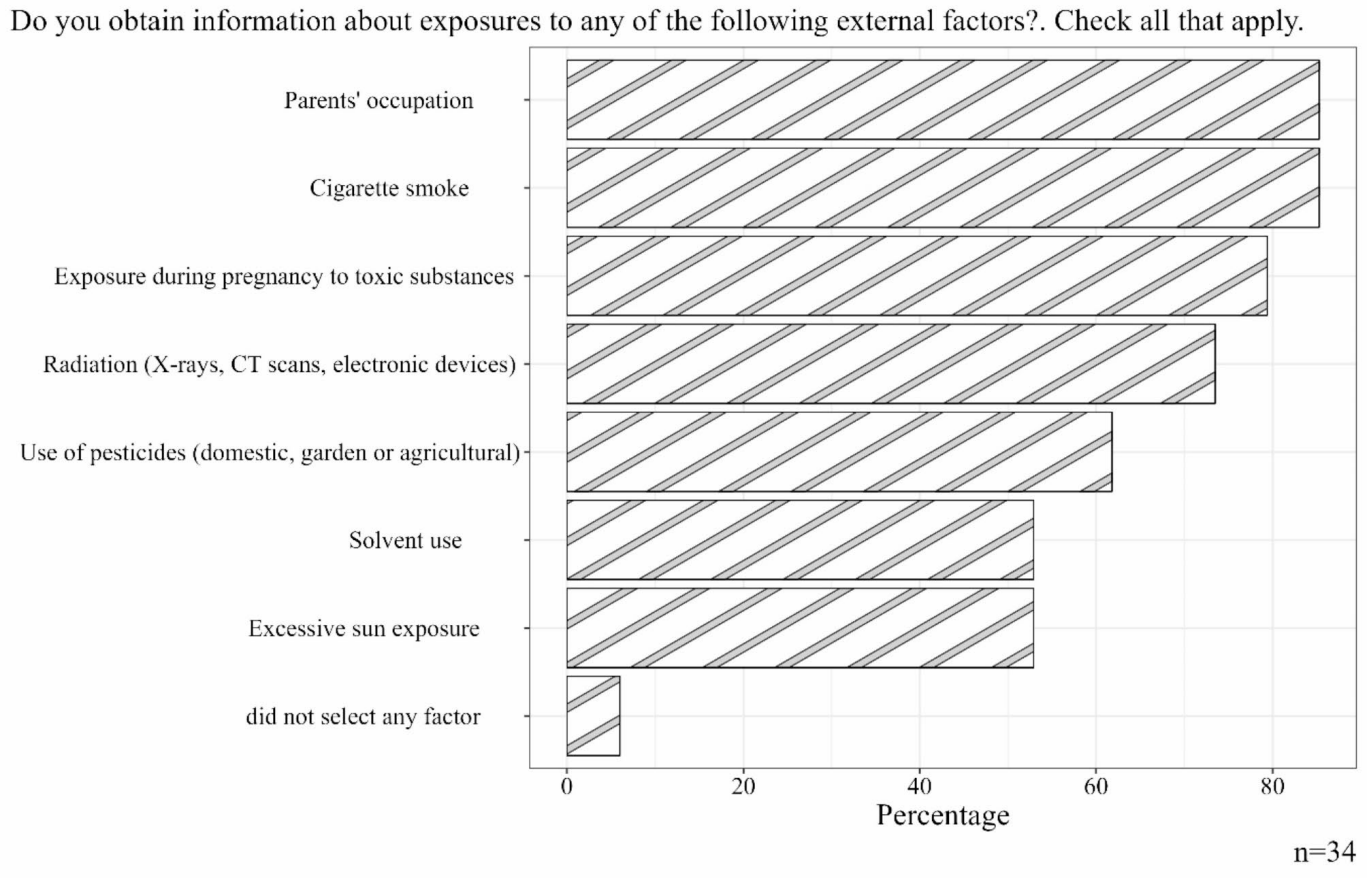
Distribution of the responses analyzed on whether medical guild obtain information on external risk factors in their medical practice

The medical guild was asked if, as part of the medical history of their patients, they obtained information on some external factors such as the parents’ occupation, cigarette smoke, exposure to toxic substances during pregnancy, radiation, use of pesticides, use of solvents and/or excessive sun exposure. 85% of them said they asked about the parents’ occupation and cigarette smoke, 79% about exposure to toxic substances during pregnancy, and 73% about radiation; between 50% and 62% of the medical guild selected the use of pesticides, solvents and excessive sun exposure (Fig. 2).

One of the questions in the survey for undergraduate students was if they had received information related to childhood cancer and suspected exposure to environmental toxins as part of their academic training. Almost 60% of the undergraduate students in medicine, 42% of those in environmental sciences and health, and 37.5% in physiotherapy responded positively to this question.

In the section to share information about the contribution of the environment to childhood cancer from their own perspective; both groups of participants wrote ideas about the importance of creating tools for cancer diagnosis including environmental risks and the need to design strategies for the control and prevention of cancer in children with the correct identification and reduction of avoidable risk, conducting more studies about the contribution of the environment to childhood cancer, encourage changes in diet and habits, communicate to society about prevention measures and spread the word on the topic.

## Discussion

We found in this research that both groups of participants consider that cancer in childhood is a disease of genetic origin,  88.2% in the medical guild group versus  97.7% in the undergraduate students group. This is indeed surprising because current data indicate that between 5 and 10% of all children with cancer have a predisposition linked to genetic factors [2, 16, 17]. Although cancer is a leading cause of mortality in children and adolescents [17] and extensive epidemiological research and international agencies have recognized the environmental chemical contribution to the cancer burden, obstacles remain in translating this evidence into action in clinical practice. The limited training in environmental medicine of medical guild, in addition to the lack of information on the concentration of carcinogens in air or water and knowledge about the sources of carcinogen emissions, reduce the possibility of correctly classifying and recording these environmental threats in medical records. Additionally, chemical profiling exposures are not requested in routine clinical testing, and some biomarkers of exposure reflect actual exposures. To carry out these analyses, expensive analytical equipment, and highly trained professionals are essential. Most data on the biological concentration of carcinogens in the general population are obtained for specific research purposes.

Environmental health sciences provide evidence about the contribution of the environment to some cancers in children, such as ALL, AML, and non-Hodgkin’s Lymphoma. Oby/Gyn and pediatric visits allow patients to start discussing cancer prevention. The main obstacle is the lack of prevention-oriented guidelines about cancer in children and environmental hazards readily available for use in clinical settings. Medical guild adequately trained in cancer and the environment are crucial to cancer prevention. Following maternal exposure, chronic low doses of carcinogens accumulate in the prenatal and postnatal period [18]. Since environmental factors require a considerable exposure time to present their carcinogenic effects, these go unnoticed and, in most cases, are not taken into account to establish childhood cancer risk scenarios. The medical guild should advise pregnant women and parents or caregivers to eliminate individual behaviors and identify avoidable or modifiable environmental risk factors, taking advantage of the fact that a child visits their pediatrician more times than an adult visits their doctor [19].

The flexibility or lack of policies to regulate and control emissions of environmental carcinogens endangers children’s health and the opportunity to prevent cancer at the primary level of care. Therefore, prevention must be supported through education and continuous training of medical guild about cancer and the environment, considering that reducing environmental risks could prevent 1 in 4 childhood deaths [20]. In the survey we applied, 85% of medical guild participants stated that they obtain information from parents for the children’s medical history about parental occupation, exposure to cigarette smoke, and exposure to toxic substances during pregnancy (79%). The first two questions can be answered with precision, but exposure to toxic substances is more challenging. General population is unaware of the types and concentrations of chemicals exposed in air, water or food. Furthermore, it is difficult to link the sources of hazardous pollutants emissions to the name of the pollutants, so the real threat is unknown. To help populations understand, reduce, or eliminate environmental risks, the medical guild requires training in environmental health that allows them to acquire the expertise of researcher and risk communicator [21–25].

Nowadays, more than 100 diseases are related to environmental pollutants [26], and healthcare professionals do not have academic training in health-environment to face these problems [24, 27–29]. This situation of vulnerability makes it difficult for the medical guild to address environmental issues with his patient, perhaps for this reason more than 90% of respondents in this study responded that it would be useful for them to have more knowledge about health and the environment. This situation is similar to that recorded in a survey of physicians and nurses in which more than 90% of respondents believed that a better understanding of the associations between environmental exposures and childhood cancer would be useful in addressing these issues with their patients [27].

In this study, both medical guild and undergraduate student participants expressed in the comments section the importance of creating tools for the diagnosis of cancer and measures to control or prevent it from its origin and assertively communicate the risks to patients. A recent study reported a low percentage of courses on environmental health in Latin American countries, including Mexico, concluding that it is necessary to reduce the gap between human and environmental health through improvement of the training of health professionals [28]. In the US, Canada and Spain, there are Pediatric Environmental Health Specialty Units (PEHSUs) that use the environmental clinical history to record environmental exposures and apply the precautionary principle in clinical practice [21, 23, 29]. In Mexico there are institutions related to the environmental health sector that are part of hospitals and universities [30]. However, these Mexican institutions need to consolidate the objectives of their approach and join efforts to link health with the environment. An environmental carcinogens pediatric clinical history must be developed to identify critical exposures that could be avoided [21, 31]. This study has limitations; the results do not represent the whole population since participation was from a non-probabilistic sample. However, knowing about the perception and attitudes through this pilot study of the medical guild and undergraduate students on the contribution of the environment to childhood cancer offer a valuable opportunity to set a baseline to know the level of knowledge in the field of cancer and environment in Mexico.

## Conclusions

In this study, the environmental contribution to childhood cancer is not clear among the medical guild and undergraduate students. They should be trained on the topic of cancer and the environment.

## Data Availability

The datasets used and/or analyzed during the current study are available from the corresponding author upon reasonable request.

## Declarations

In this investigation, informed consent was obtained from all participants and all methods were carried out in accordance with the relevant guidelines and regulations of the Declaration of Helsinki.

## Abbreviations

ALL: Acute Lymphoblastic Leukemia
AML: Acute Myeloid Leukemia
CIAAS: Center for Applied Research in Environment and Health
US: United States
PEHSUs: Pediatric Environmental Health Specialty Units
